# Strong nutrition governance is a key to addressing nutrition transition in low and middle-income countries: review of countries’ nutrition policies

**DOI:** 10.1186/1475-2891-13-65

**Published:** 2014-06-27

**Authors:** Bruno F Sunguya, Ken IC Ong, Sumi Dhakal, Linda B Mlunde, Akira Shibanuma, Junko Yasuoka, Masamine Jimba

**Affiliations:** 1 Department of Community and Global Health, Graduate School of Medicine, The University of Tokyo 7-3-1, 113-0033 Hongo Bunkyo-ku, Tokyo, Japan

**Keywords:** Nutrition transition, Low and middle-income countries, Undernutrition, Overweight, Obesity

## Abstract

**Methods:**

We reviewed nutrition policies, nutrition governance, and the trends of nutrition status from LAMICs. We retrieved data on the policies from the global database on the implementation of nutrition actions (GINA). We also retrieved data on the trends of nutrition status and nutrition governance from the nutrition landscape information system (NLiS), and on LAMICs from the World Bank database. We then analyzed the data both descriptively and by using a mixed effects model with random-intercept by country.

**Results:**

Of the 139 LAMICs reviewed, only 39.6% had policies to address both undernutrition and overweight/obesity. A higher proportion of low-income countries (LICs) had policies to address undernutrition compared to that of middle-income countries (MICs) (86.1% vs. 63.1%, p = 0.002), and a low proportion of both had policy to address overweight. Having a nutrition policy that addresses undernutrition was not associated with better nutrition status outcomes. Strong nutrition governance in LAMICS was associated with low magnitudes of stunting (beta = -4.958, p = 0.015); wasting (beta = -5.418, p = 0.003); and underweight (beta = -6.452, p = 0.001).

**Conclusion:**

Despite high magnitudes of undernutrition and overweight/obesity in LAMICs, only about one third of them had nutrition policies to address such nutrition transition. Having strong nutrition governance may help to bring down the magnitudes of undernutrition in LAMICs.

Nutrition transition necessitates low and middle-income countries (LAMICs) to scale up their efforts in addressing the burdens of undernutrition and overweight/obesity. Magnitudes of undernutrition and overweight are high in LAMICs, but no study has reviewed the existence of nutrition policies to address it. No evidence is also available on the effect of nutrition policies and governance on the undernutrition and overweight/obesity patterns in such countries. We conducted a policy review to examine the presence and associations of nutrition policies and governance with the children’s nutrition statuses in LAMICs.

## Background

The burden of undernutrition is slowly decreasing in developing countries [1]. However, the rate of this decline is slow and a new threat of overweight and obesity in these countries is beginning to become evident [1,2]. This unprecedented trend is due to the shift in feeding practices and energy expenditure that coincides with economic development, demographic transition, and epidemiological changes in the population [3,4], also known as nutrition transition.

Low and middle-income countries have historically suffered from a high burden of child undernutrition, predominantly due to food insecurity, socio-economic and demographic disadvantages, high burdens of infectious diseases, and other biological and social determinants [1]. Rapid economic development and technology [5,6] have helped to increase food production and generated surplus. The growing necessity of urban migration [6,7] together with demographic transition [8], and less physical activity in increasingly sedentary lives are behind the rising magnitudes of overweight among adults and children in these countries [7,9].

Increasing economic disparity and inequality in low and middle-income countries has led to food insecurity for some people, and food abundance with sedentary lifestyles in others. As a result, the problem of undernutrition is still rampant yet at the same time magnitudes of overweight and obesity are also increasing, even in the same communities [2]. Such trends are particularly common among children [1,10]. The end results of such nutritional transitions have already become evident in high-income countries, with unprecedented rates of overweight and obesity but greatly reduced proportions of undernutrition [11,12]. This necessitated nutrition policies and actions to address growing rates of overweight and obesity, which are helping to bring down rates of non-communicable disease in high-income countries [11].

Transition from undernutrition into coexistence of undernutrition and overweight/obesity is an important cause of the increasing burden of non-communicable diseases (NCDs) [9,12]. Children born with low weight or those who succumb to undernutrition in their early childhood have a high risk of early adulthood obesity and NCDs, including diabetes and heart diseases [12-15]. In this regard, countries where undernutrition is rampant may have high magnitudes of early adulthood obesity and subsequent non-communicable diseases [16]. Currently, low and middle-income countries contribute 70% of all NCD-related mortality globally [2]. Even without the added burden of childhood undernutrition as a precursor, these countries have other high risk factors for NCDs, including low levels of physical activity and unhealthy eating. These risk factors alone are responsible for 5% and 6% of global deaths, respectively [2], and are unacceptably high in LAMICs.

Interventions are known to combat child undernutrition [17,18]. Some of them include infant and young child feeding intervention, with exclusive breast feeding and proper weaning; proper feeding practices at an adequate frequency and diversity; maintain hygiene and prevention of diarrhea; and micronutrient supplementation including vitamin A. A combination of interventions is also important to address risk factors for overweight and obesity [2,19]. A balance of interventions for both undernutrition and overweight based on a country’s situation can help to address both burdens [10]. Such efforts should be stipulated in a country’s nutritional policy and integrated into a national health policy and plan [20]. However, efforts are lagging behind on policies to address overweight as compared to undernutrition in developing countries [16,21-23]. Lack of updated nutrition policies and poor nutrition governance to address such dual burdens in developing countries are a cause of this status quo [24]. As a result, slow achievements are being made on undernutrition [1], while no progress or even a worsening trend can be seen for overweight and obesity [1,2].

Despite the importance of updating countries’ nutrition policies to reflect the nutrition transition [25], no study has reviewed the presence of such policies. Evidence is also lacking on the potential effects of nutrition policies to address trends in nutrition status including stunting, underweight, wasting, and overweight/obesity. Nutrition policy may need accompanying country-level nutrition governance in order to implement nutrition related interventions and achieve the desired output. In this aspect too, evidence is lacking. We therefore conducted this review to examine whether low and middle-income countries have nutrition policies to address the burdens of undernutrition and overweight/obesity, and the association of such policies and nutrition governance on magnitudes of undernutrition and overweight.

## Methods

We reviewed data from LAMICs to examine the presence of nutrition policies that can address the dual burdens of undernutrition and overweight/obesity. In this review, we also aimed to examine whether having such policies and whether having stronger nutrition governance may be associated with low magnitudes of undernutrition and overweight among under-five year old children.

### Novelty checks and protocol development

We conducted a novelty check before this review to determine what existing studies have been conducted on nutrition policies and to identify research gaps. Two reviewers (BS and LM) searched for the presence of similar topics, published protocols, and ongoing and published reviews in relevant databases. Such databases included the Cochrane Database of Systematic Reviews (CDSR), Database of Abstracts of Reviews of Effects (DARE), and Educational Resources Information Center (ERIC). We developed a review protocol and registered it at the Center for Reviews and Dissemination (CRD) database [26].

### Databases, variables, and data extraction procedures

The current review used data from two World Health Organization (WHO) nutrition databases and the World Bank database. The nutrition databases were the Global database on the Implementation of Nutrition Actions (GINA) and the Nutrition Landscape Information System (NLiS).

Three reviewers (BS, KO, SD) independently extracted data from the three databases based on the registered protocol [26]. We used the World Bank dataset to select countries that fit the definition of LAMICs [27].

#### Nutrition status

Nutrition status was the main outcome variable of interest in this review. It was measured as undernutrition or overweight status. For undernutrition we extracted the data on countries’ prevalence of stunting, wasting, and underweight. Stunting was measured as the percentage of children whose height for age was below minus 2 standard deviations of the WHO child growth standards median [25,28]. Underweight was measured as the percentage of those children whose weight for age was below minus 2 standard deviations of the WHO child growth standards median [25,28]. Wasting was defined and the percentage of children whose weight for height was below minus 2 standard deviations of the WHO growth standards median. Overweight was defined as the percentage of children whose weight for age was above plus 2 standard deviations of the WHO child growth standards median [25,28].

Wasting and underweight reflect acute growth faltering. Underweight is less specific on timing but wasting indicates severe acute undernutrition [29,30]. Both can result from severe shortage of food, diseases, or conditions that cause acute loss of weight such as diarrhea and acute febrile illnesses. Stunting on the other hand, reflects a more cumulative retardation of growth mainly due to chronic causes, such as poor diet, chronic hunger, and recurrent or chronic infections [29,30]. Child overweight results from poor feeding practices and physical inactivity. It is associated with obesity and high risk of non-communicable diseases early in adulthood [1,14].

We extracted data about the prevalence of each type of nutrition status for each of all the LAMICs from the NLiS [25]. The NLiS [25] is managed by the WHO under the department of nutrition for health and development. It contains data that were made publicly available from the WHO, United Nations Children’s Fund (UNICEF), the UN statistics division, the United Nations Development Programme (UNDP), the Food and Agricultural Organization (FAO), Demographic and Health Surveys (DHS), the World Bank, the International Food Policy Research Institute (IFPRI), and the International Labor Organization (ILO). Such different sources could however lead to differences in measurements, reporting, and reference standards. NLiS has countries’ data on child undernutrition including stunting, wasting, underweight, and overweight. Such data are available for different year intervals, making it easier to assess trends. We therefore extracted three sets of anthropometric data and the years they were collected. For example, one country may have three different data on stunting at three different years, and we extracted all of them. Moreover, NLiS also has information on countries’ nutrition governance index and the years of their assessment.

#### Nutrition policies

One of the main independent variable in this study was nutrition policy. In this review, we examined whether a country had a nutrition policy that addressed undernutrition or overweight/obesity. We included policies that address underweight as those that focus on underweight, stunting, and wasting. Policies that addresses micronutrient deficit alone were not included, as the focus of this review was on nutrition transition. Moreover, we did not include policies that address outcomes of poor nutrition status alone without the nutrition status of interest. For a policy to be inclusive, and targeting majority of populations in each country, we selected those originating from the government and/or ministry of health for this review. International and non-governmental organization driven policies were not included in this review.

We extracted nutrition policies from the GINA database [31]. Nutrition policies and actions in GINA were national policies; international organizations’ initiated/implemented policies; and non-governmental organization-run nutrition policies. It also lists legislations addressing nutrition related issues, years in which they were formulated and adopted, and whether they are targeting the nationwide population or specific groups. We extracted data on nationwide nutrition policy as the mainstay policy. We created two variables from the list of policies available for each country. The first was whether the country had any policy that addresses the problem of undernutrition (wasting, underweight or stunting). The second variable was whether the country had any policy that addresses the problem of overweight or obesity. Then, we made a variable of nutrition policy in effect, so as to match the presence or absence of nutrition policy during the anthropometric measurement. This was done to see the association of presence or absence of the policy on nutrition status.

#### Nutrition governance

Nutrition governance was one of our independent variable. It describes the strengths and weaknesses of various aspects of nutrition activities in a given country [25]. This scale was made of ten elements: existence of an intersectoral mechanism to address nutrition; existence of a nutrition strategic plan; whether the strategy is adopted; whether the strategy is part of the national development plan; existence of a national nutritional policy; whether the policy is adopted; existence of dietary guidelines; allocation of the budget for implementation of the national nutrition plan, strategy, or policy; regular monitoring and surveillance; and existence of a nutrition component in the health budget [25]. Having a nutrition policy is one of the components to make strong nutrition governance, but it is not enough, and cannot act alone. Nutrition governance is scored as strong, medium, or weak. The nutrition governance was coded as 1 = weak; 2 = medium; and 3 = strong, and entered into the regression analyses as a categorical variable.

We used the NLiS database [25] to extract information about the country’s nutrition governance. A few countries had been evaluated and ranked for nutrition governance (Additional file 1: Table S1). According to the database and its manual, nutrition governance was measured between 2008 and 2009.

#### Country’s income level

World Bank classifies countries according to their income level. This review focused on LAMICs. They are also sub-divided into selected Low Income Countries (LICs) and Middle Income Countries (MICs). MICs are further classified into Lower Middle Income Countries (LMICs) and Upper Middle Income Countries (UMICs). Income levels of each country can predict the level of undernutrition or overweight. We therefore treated this as a confounding variable in this review. We extracted a list of countries and their development indices from the World Bank database [27]. A recent classification gave a total of 139 LICs and MICs (Additional file 1: Table S1).

#### Gross domestic product per capita

Gross Domestic Product (GDP) per capita of each country may affect nutrition policies, nutrition status, and nutrition governance. We therefore included GDP per capita as one of the confounding variables. GDP per capita is the sum of gross value added by all resident producers in the country including product taxes and minus subsidies out of the value of the products [32]. We extracted data of GDP per capita for each LAMIC from the World Bank database. We matched years of GDP per capita data and those of data on nutrition status for each country.

#### Averaged aggregated governance indicator

Nutrition and health of population may be associated with other structural indices including political situation, country’s governance, among others. In this study, we included aggregated governance indicator as a confounding variable. The averaged aggregate governance indicator represents the aggregated average of the six world governance indicators made by the World Bank [25]. The six indicators are aggregated and using z-scores, the scale that ranges from -2.5 to +2.5 was made. Higher score represents higher averaged aggregated governance indicator.

In the current review, we extracted the data of averaged aggregated governance indicator from the NLiS database. It was treated as a continuous variable to preserve small differences between nations.

#### Net primary school enrolment

Education is an important determinant of health. Levels of education of caregivers may also affect nutrition status and therefore a confounding variable in the current review. In this study, we selected the country’s net percentage of primary school enrollment as it represents the ratio of official primary school aged children who enrolled in primary school to the total population of the official primary school aged children. It can give a proxy measure of the basic education level in a given country. We extracted this variable from the updated World Bank database and treat it as a continuous variable. To examine its effect on nutrition status, we collected data of school enrolment for years that data on nutrition status was available.

### Analyses

We analyzed the data both descriptively and using multivariate analyses. For descriptive analyses, we used both the chi-square test and the student’s *t*-test. We also used Fisher’s exact test to describe years of nutrition policies, using 10 years as a cut-off, in LIC and MICs. The 10 years was arbitrary chosen based on the average time taken to update a policy as observed in GINA. To examine the presence of nutrition policies in LICs and MICs, we used the chi-square test. We compared presence of a policy that address undernutrition and overweight separately, stratified by the country’s income level. We used the student’s *t*-test to compare mean rates of stunting, wasting, underweight, and overweight by country’s level of income.

We conducted regression analyses using mixed effects models with random-intercept by country, to take into account differences each country has. We built four models using each type of nutrition status as an outcome variable. These were stunting, wasting, underweight, and overweight. We had two main independent variables; nutrition policy and nutrition governance. For nutrition policy, we coded as 1 if a country had nutrition policy to address undernutrition or overweight that was in effect at each anthropometric measurement year. We used undernutrition policy for models that had undernutrition as an outcome variable and overweight policy for model with overweight status as the outcome variable. A similar method was used to generate nutrition governance variable that was in effect at time of anthropometric data collection.

The main confounding variables included GDP per capita, net percentage of primary school enrolment, and country governance. We matched years of data of GDP per capita and net school enrolment with years of anthropometric measurements to control for the potential effect each had on the outcome variables. We also controlled for the year of anthropometric measurement to observe changes of the outcome variables with time.

We set the statistical significance at *p-value* < 0.05 and conducted all analyses using STATA version 12.

## Results

A total of 139 countries were included in this review. Based on the World Bank database, a total of 36 of the included countries were classified as LICs and 103 as MICs. Of the total MICs, 48 were LMICs while 55 were classified as UMICs Additional file 1: Table S1.

### Prevalence of undernutrition and overweight/obesity among LICs and MICs

Table 1 shows the results for the mean values of countries’ prevalence of types of nutrition status stratified by their development index. The most recent data on stunting was available for 104 countries. Of them, 32 were LICs and 72 MICs. Compared to MICs, a higher percentage of children in LICs were stunted (39.5 vs. 23.6, p < 0.001). Wasting was also more prevalent among children in LICs compared to those of MICs. Of the 102 countries with the most recent data on wasting, 31 were LICs. On average, 8.6% of the children in these countries were wasted, compared with 5.9% of those from MICs, p = 0.008. Also, compared to MICs, underweight was more prevalent among children in LICs (21.8% vs. 11.1%, p < 0.001). On the other hand, more children in MICs were overweight compared to those of LICs (8.8% vs. 4.9%, p < 0.001).

**Table 1:** Nutrition status of children underfive stratified by countries development index (Low income vs. middle income)

**% Nutrition status**	**Low income countries**			**Middle income countries**			**p-value**
	**N**	**Mean %**	**S.D.**	**N**	**Mean %**	**S.D.**	
**Stunting (N = 104)**	32	39.5	8.2	72	23.6	12.0	< 0.001
**Wasting (N = 102)**	31	8.6	4.1	71	5.9	4.8	0.008
**Underweight (104)**	31	21.8	8.6	73	11.1	10.2	< 0.001
**Overweight (96)**	30	4.9	4.1	66	8.8	4.2	< 0.001

Across the countries, values for kurtosis for Stunting = 2.3; Wasting = 3.4; Underweight = 3.1; and Overweight = 3.

Table 2 shows the magnitudes of undernutrition in LAMICs by the regions. All forms of undernutrition were more prevalent in LAMICs of South Asian region compared to others. In the LAMICs of this region, stunting was 38.2%, wasting 12.3%, and underweight 28.2%. LAMICs of Eastern Europe and central Asian region had the lowest magnitude of stunting (14.6%) and underweight (4.0%) but had the highest magnitude of overweight (14.2%).

**Table 2:** Regional distribution of undernutrition and overweight and nutrition policies to address them among underfive children in low and middle-income countries

**Region**	**Countries n (%)**	**Stunting %**	**Wasting %**	**Underweight %**	**Overweight %**	**Nutrition policy undernutrition**	**Nutrition policy overweight**
**South Asia**	8 (5.8)	38.2	12.3	28.2	3.9	8 (100.0)	3 (37.5)
**Eastern Europe & Central Asia**	20 (14.3)	14.6	4.0	4.0	14.2	7 (35.0)	9 (45.0)
**Middle East and North Africa**	13 (9.4)	22.0	7.6	10.3	12.2	8 (61.5)	5 (38.5)
**East Asia and Pacific**	24 (17.3)	27.1	6.9	15.2	5.9	16 (66.7)	12 (50.0)
**Sub Saharan Africa**	47 (33.8)	35.4	8.9	19.5	6.3	38 (80.9)	22 (46.8)
**Latin America and Caribbean**	27 (19.4)	18.4	2.6	5.3	7.2	19 (70.4)	12 (44.4)
**Total (%)**	139 (100.0)	27.0	6.8	13.8	7.8	96 (69.1)	63 (45.3)

### Presence of nutrition policy to address the burdens of undernutrition and overweight/obesity

Table 2 and Additional file 2: Table S2 show the results of the presence of nutrition policy to address undernutrition or overweight/obesity by regions. Of 139 LAMICs, 69.1% and 45.3% had policy to address undernutrition and overweight, respectively. All eight South Asian LAMICs had policy to address undernutrition, but the lowest proportion of them (37.5%), had policy to address overweight/obesity. Half of LAMICs in East Asia and Pacific had policy to address overweight and more than two thirds of them had policy to address undernutrition. In Sub Saharan region, 80.9% and 46.8% of LAMICs had policy to address undernutrition and overweight, respectively.

Table 3 shows distribution of presence of nutrition policies and governance stratified by country’s level of development. A total of 31 out of 36 LICs had nutrition policy to address undernutrition, a higher proportion compared to that of MICs (86.1% vs. 63.1%, p = 0.002). More than half of both LICs (55.6%) and MICs (54.4%) had no nutrition policy to address overweight and obesity. A total of 55 out of 139 LICs and MICs had policies that address the burdens of both undernutrition and overweight/obesity. This translates to only 39.6% of all countries under this income level. Of them, 16 (44.4%) were LICs and 39 (37.9%) were MICs. The difference between them did not reach a significant level (p = 0.487).

**Table 3:** Presence of a nutrition policy and governance to address undernutrition and overweight among children underfive in low and middle-income countries

	**Low income countries (n = 36)**		**Middle income countries (n = 103)**		**p-value**
	**n**	**%**	**n**	**%**	
**Nutrition policy for undernutrition (n = 139)**					
**Yes**	31	86.1	65	63.1	0.002
**No**	5	13.9	38	36.9	
**Nutrition policy for overweight/obesity (n = 139)**					
**Yes**	16	44.4	47	45.6	0.902
**No**	20	55.6	56	54.4	
**Nutrition policy for Undernutrition and Obesity (n = 139)**					
**Yes**	16	44.4	39	37.9	0.487
**No**	20	55.6	64	62.1	
Nutrition policy within 10 years for undernutrition (n = 96)*					
**Yes**	28	90.3	49	75.4	0.070
**No**	3	9.7	16	24.6	
**Nutrition policy within 10 years for overweight/obesity (n = 63)**					
**Yes**	16	100.0	42	89.4	0.218
**No**	0	0.0	5	10.6	
**Nutrition governance (n = 36)**					
**Weak**	8	47.1	5	26.3	0.196
**Moderate or strong**	9	52.9	14	73.7	

*Fisher’s exact test.

About one quarter of MICs with nutrition policies to address undernutrition had developed the policies more than ten years ago. This compares with less than one tenth of the policies present in LICs being developed more than ten years ago. Also, although only 16 LICs out of 31 had policies to address overweight and obesity, all were made within the past ten years. Among MICs, five had policies to address overweight and obesity that were developed more than ten years ago (Table 3).

### Nutrition governance of LICs and MICs

Table 3 also shows the results for nutrition governance stratified by country’s development level. A total of 36 countries had data on nutrition governance. Of them, 17 were LICs, of which half had weak nutrition governance. By comparison, only five out of 19 MICs had weak nutrition governance. Of all countries with strong nutrition governance, 69.6% had a policy to address overweight compared to only 38.5% of countries with weak nutrition governance (p = 0.069) (Table 4). No significant association was found for policies to address undernutrition.

**Table 4:** Bivariate association between nutrition governance and nutrition policy to address children’s undernutrition and overweight

**Nutrition policy**	**Weak nutrition governance**		**Strong nutrition governance**		**p-value**
	**n**	**%**	**n**	**%**	
**Undernutrition**					
**Yes**	12	92.3	21	91.3	0.917
**No**	1	7.7	2	8.7	
**Overweight/obesity**					
**Yes**	5	38.5	16	69.6	0.069
**No**	8	61.5	7	30.4	

### Association of nutrition policies and governance with changes of stunting, wasting, and underweight statuses among LICs and MICs

Table 5 shows the results for the mixed effects model with random-intercept by country to examine the associations of nutrition policy and nutrition governance with nutrition statuses. A total of 104, 102, 104, and 96 out of 139 LAMICs had data on stunting, wasting, and underweight, respectively. A total of 34 LAMICs had data on stunting and nutrition governance. Thirty-six had data on underweight and nutrition governance, 34 on wasting and nutrition governance, and 31 on overweight and nutrition governance.

**Table 5:** Association of nutrition policy and nutrition governance with county’s undernutrition status of children underfive in low and middle-income countries

	**Stunting status**			**Wasting status**			**Underweight status**		
**Variable**	**Coefficient**	**95% CI**	**P**	**Coefficient**	**95% CI**	**P**	**Coefficient**	**95% CI**	**P**
**GDP per capita**	-0.003	-0.005 − -0.002	<0.001	-0.001	-0.002 − 0.001	0.276	-0.002	0.003 − -0.001	0.014
**School enrolment**	-0.171	-0.290 − -0.054	0.004	-0.162	-0.236 − -0.088	<0.001	-0.264	-0.349 − -180	< 0.001
**Country governance**	-1.890	-9.934 − 6.154	0.645	-2.398	-6.097 − 1.300	0.204	-9.552	16.707 − -2.399	0.009
**Trend (year)**	0.104	-0.422 − 0.630	0.279	0.279	-0.085 − 0.642	0.133	0.112	-0.272 − 0.496	0.566
**Policy in effect**	-2.608	-6.252 − 1.037	0.161	1.834	-0.726 − 4.395	0.160	-0.466	-2.865 − 1.932	0.703
**Nutrition governance**									
**Weak**	0.859	-4.171 − 5.889	0.738	-0.892	-4.735 − 2.952	0.649	-1.443	-4.697 − 1.810	0.385
**Medium**	2.990	-2.213 − 8.190	0.260	1.498	-2.681 − 5.681	0.482	2.117	-1.178 − 5.411	0.208
**Strong**	-4.958	-8.970 − -9.460	0.015	-5.418	-8.936 − -1.900	0.003	-6.452	-8.937 − -3.967	0.001

P = p-value, GDP = Gross domestic product, CI = Confidence interval.

Every one-dollar increase in per capita GDP was associated with a 0.003-unit decrease in stunting (p < 0.001) and a 0.002-unit decrease in underweight (p = 0.014). A one-percent increase in net school enrolment in LAMICs was more likely to be associated with a 0.171-unit decrease in stunting (p = 0.004); a 0.162-unit decrease in wasting (p < 0.001); and a 0.264-unit decrease in underweight (p < 0.001). An increase of one unit in the aggregate country governance indicator correlated with a 9.552-unit decrease in underweight (p = 0.009). There was no significant decrease for the prevalence of undernutrition over years among LAMICs after adjusting for key confounders.

The associations between presence of a nutrition policy to address undernutrition in effect and magnitudes of each type of undernutrition over time did not reach a statistically significant level. However, an increase from weak to strong nutrition governance in effect was more likely to correlate with a 4.958-unit decrease in stunting (p = 0.015); a 5.418-unit decrease in wasting (p = 0.003); and a 6.452-unit decrease in underweight (p = 0.001).

A total of 96 countries had data on the overweight status among 139 LAMICs. Table 6 shows the results for the mixed effects model with random-intercept by country to examine changes in overweight prevalence as a result of the presence of nutrition policy and nutrition governance. Contrary to undernutrition, an increase in every one-dollar in GDP per capita was more likely to be associated with a 0.001-unit increase in overweight (p = 0.002). Having a policy to address overweight in effect was not associated with decrease in the prevalence of overweight. Although a strong governance and overweight status in LAMICs showed an inverse relationship, such association did not reach a significant level (beta = -3.755, p = 0.061).

**Table 6:** Association of nutrition policy and governance with county’s overweight status of children underfive in low and middle-income countries

**Variable**	**Coefficient**	**95% CI**		**p-value**
		**Low**	**High**	
**GDP per capita**	0.001	0.001	0.002	0.002
**School enrolment**	0.010	-0.056	0.076	0.764
**Country governance**	1.428	-1.912	4.767	0.402
**Trend (year)**	-0.028	-0.368	0.311	0.870
**Policy in effect**	1.909	-1.157	4.974	0.222
**Nutrition governance**				
**Weak**	0.206	-3.175	3.588	0.905
**Medium**	-0.689	-4.483	3.104	0.722
**Strong**	-3.755	-7.689	0.180	0.061

## Discussion

A total of 139 low and middle-income countries (LAMICs) were included in this review. Of them, 36 were low-income countries (LICs), 48 were lower-middle income countries (LMICs), and 55 were upper-middle income countries (UMICs). As also reported previously [1,10,33], LICs had a higher average prevalence of all types of undernutrition but lower prevalence of overweight compared to MICs. Regionally, undernutrition was more prevalent in South Asia compared to other regions. In contrast, overweight was more prevalent among LAMICs of Eastern Europe and Central Asia, but undernutrition was in the lowest magnitudes in this region compared to others. Despite the high magnitudes of undernutrition and overweight/obesity in LAMICs, only 37.9% had nutrition policies that could address the dual burdens. Moreover, of the 36 countries with available data, a higher proportion of LICs had weak nutritional governance compared to MICs. This study also found that strong nutrition governance is associated with lower magnitudes of undernutrition.

Like in previous studies, our study found that all undernutrition statuses were more prominent in LICs than in MICs [1,10,33]. Higher prominence of undernutrition in LICs than MICs is likely due to the predominance of the determinants of undernutrition [34,35]. These include food insecurity, poor feeding practices, high burden of diseases, and other socio-economic disadvantages which are more common in poor settings [35]. On the other hand, food abundance, sedentary lifestyles and urbanization have been responsible for the opposite extreme of nutrition—overweight and obesity, a trend that is on the rise in MICs [9,10]. In addition, an increase in GDP per capita was associated with a decrease in the undernutrition prevalence while it was associated with an increase in prevalence of overweight. Also, such economic development may bring about increase in the number of educated caregivers who can impact children nutrition. We found an association between net school enrolments with the reduction of the prevalence of undernutrition. With rapid economic development and demographic transition, the magnitudes of undernutrition tend to improve [36]. At the same time, populations that are well off and affluent, move to the other extreme end of nutrition statuses. During this process rates of overweight and obesity go up, but tend to continue to coexist with high levels of undernourishment-a phenomenon best described as nutrition transition [22].

Nutrition transition is evident in LAMICs. This study found a persistent magnitude of undernutrition that coexists with those of overweight in these countries. Despite such finding, only 37.9% of the countries had nutrition policies that could address both burdens. Without a nutrition strategy outlined in a country’s nutrition policy and adopted in overall health policy, efforts to control undernutrition may not reach the majority of the population and any efforts may yield poor outcomes [24,25]. The majority of LICs had policies to address only undernutrition. This is clearly because of a historical concern over undernutrition in such countries [35]. Under such settings, undernutrition was regarded as a problem of public health importance. A little is done for overweight, a condition that is also growing in such countries. With such growing threat, it is high time for these countries to include overweight in their health policies and national strategies.

This study found no significant association between nutrition policy to address undernutrition or overweight with reduction in the magnitudes of either undernutrition or overweight in LAMICs. Having policy alone may not be enough to bring down rates of undernutrition and overweight/obesity [24]. To enact a policy, a country would need stewardship, adopting the policy and streamline it with national development agenda, having a nutrition strategic plan, funding for interventions included in the policy, and involving other sectors, in this case, a strong nutrition governance [25]. Although nutrition policy is one component in the nutrition governance [25], to develop, enact, and initiate nutrition action, other components of nutrition governance are also important. Together, such components can bring about the desired change [37]. Governance is also important for showing how a country is committed to accelerating nutrition actions [38,39]. It is a measure of the country’s responsiveness to varied threats of nutrition, both undernutrition and obesity [38]. In this study, MICs had stronger nutrition governance compared to LICs. Results of this study further showed that strong nutrition governance was more likely to be associated with improved magnitudes of undernutrition and showed a direction towards improving magnitudes of overweight, after controlling for other confounding variables.

Results of this study should be discussed in light of the following potential limitations. First, this study reviewed data whose sources might have used different methods and tools for collection. For example, we used nutrition status data, which is reported by the WHO, but originated from national sources, DHS, and other UN organizations. Although this could have lead to over- or under-estimation, these are the best estimates available and have been adopted globally. Second, to examine the changes of nutrition statuses over time, we used data for as many different years and different countries as we could get. Such analyses may have been more accurate if data for the same time intervals and years was available. To mitigate the effect of time differences, we controlled for the years in the regression analyses. We also collected data of GDP per capita and school enrolment that corresponded to the years of nutrition status data collection. We controlled nutrition policy and governance by matching them with years of anthropometric data. Third, only a few countries had data on nutrition governance. This could reduce the power of our results. Fourth, a few countries had data on nutrition governance, which was also our important independent variable. This might lead into under estimation of the association between it and nutrition statuses, or limit generalizability into countries with no such data. However, for the available data, we controlled important confounders to find independent association with nutrition statuses. Fifth, we used GINA database to collect data on nutrition policy. This database uses available policies in the country or ones that are provided upon request, in native languages. This may result in missing some policies or updates thereof. Sixth, while the problem of nutrition transition affects populations across age groups, we focused on child population. This was mainly because of the lack of nutrition data among adults in LAMICs and unclear nutrition policy to address such problems.

Despite the mentioned limitations, this study serves as the first to review nutrition policies to address the growing concern of nutrition transition in low and middle-income countries. It is also the first to examine the association of such policies and governance with the changes of undernutrition and overweight or obesity magnitudes.

In conclusion, this review found a low proportion of low and middle-income countries had nutrition policies to address both undernutrition and overweight/obesity. A higher proportion of LICs had nutrition policies to address undernutrition but not overweight and obesity compared to MICs. Presence of nutrition policy, however, was not associated with reduction in magnitudes of undernutrition or overweight. To ameliorate the threat of failing to address the challenges of the nutrition transition, stronger nutritional governance is necessary which is supported by updated policies that respond to the dynamic nutrition situation pertinent to a country setting. Low and middle-income countries should target both undernutrition and growing rates of overweight and obesity even if they are not yet of public health importance. Without having stronger nutrition governance, policies alone may not be enough to address the growing threat of nutrition transition.

## Competing interests

All authors read and approved the final manuscript.

## Authors’ contributions

BFS conceived the research questions, prepared the protocol, designed the study, conducted data collection, analyses, and prepared the first draft. KO conducted data collection, analyses, and prepared the first draft. SD conducted data collection. LBM helped to prepare the first draft. AS helped to conduct analyses. JY participated in the preparation of the first draft and revisions. MJ reviewed the study protocol and manuscript, supervised data collection, and approved the submission. All authors read and approved the final version of the manuscript for submission.

## Supplementary Material

Additional file 1: Table S1 Selected countries, development indices, and nutrition policies. Click here for file

Additional file 2: Table S2 Data on country, regional distribution, policy, nutrition status. Click here for file
