# Supplementary material for: Strong nutrition governance is a key to addressing nutrition transition in low and middle-income countries: review of countries’ nutrition policies
Source: Nutr J. 2014 Jun 27;13:65. doi: 10.1186/1475-2891-13-65 (PMC4110371; doi:10.1186/1475-2891-13-65)
Supplement: Additional file 1: Table S1 — Selected countries, development indices, and nutrition policies. [file 1475-2891-13-65-S1.doc]

| **Additional file 1: Selected countries, development indices, and nutrition policies** | | | | | | | |
| --- | --- | --- | --- | --- | --- | --- | --- |
| SN | **Country** | **Development Index** | **Nutrition governance** | **Undernutrition nutrition policy** | **Policy for undernutrition within 10yrs** | **Overweight/obesity nutrition policy** | **Policy for obesity within 10yrs** |
| 1 | Afghanistan | LIC | Weak | Yes | Yes | No | No |
| 2 | Albania | UMIC |  | No | No | No | No |
| 3 | Algeria | UMIC |  | Yes | No | No | No |
| 4 | American Samoa | UMIC |  | No | No | No | No |
| 5 | Angola | UMIC | Weak | No | No | No | No |
| 6 | Argentina | UMIC |  | Yes | No | No | No |
| 7 | Armenia | LMIC |  | Yes | Yes | No | No |
| 8 | Azerbaijan | UMIC |  | Yes | Yes | No | No |
| 9 | Bangladesh | LIC | Strong | Yes | Yes | Yes | Yes |
| 10 | Belarus | UMIC |  | No | No | No | No |
| 11 | Belize | UMIC |  | Yes | Yes | No | No |
| 12 | Benin | LIC |  | Yes | Yes | No | No |
| 13 | Bhutan | LMIC |  | Yes | Yes | No | No |
| 14 | Bolivia | LMIC |  | Yes | Yes | No | No |
| 15 | Bosnia & Herzegovina | UMIC |  | Yes | Yes | No | No |
| 16 | Botswana | UMIC |  | Yes | No | No | No |
| 17 | Brazil | UMIC |  | Yes | Yes | Yes | Yes |
| 18 | Bulgaria | UMIC |  | Yes | Yes | Yes | Yes |
| 19 | Burkina Faso | LIC | Strong | Yes | Yes | Yes | Yes |
| 20 | Burundi | LIC | Medium | Yes | Yes | Yes | Yes |
| 21 | Cambodia | LIC | Weak | Yes | Yes | Yes | Yes |
| 22 | Cameroon | LMIC | Medium | Yes | No | No | No |
| 23 | Cape Verde | LMIC |  | Yes | No | No | No |
| 24 | Central African Republic | LIC |  | Yes | No | No | No |
| 25 | Chad | LIC |  | Yes | Yes | No | No |
| 26 | China | UMIC |  | Yes | No | Yes | No |
| 27 | Colombia | UMIC |  | Yes | Yes | Yes | Yes |
| 28 | Comoros | LIC |  | No | No | No | No |
| 29 | Congo, Dem. Rep. | LIC | Weak | Yes | Yes | Yes | Yes |
| 30 | Congo, Rep. | LMIC |  | Yes | No | No | No |
| 31 | Costa Rica | UMIC |  | Yes | Yes | Yes | Yes |
| 32 | Cote d'Ivoire | LMIC | Medium | Yes | Yes | Yes | Yes |
| 33 | Cuba | UMIC |  | Yes | No | No | No |
| 34 | Djibouti | LMIC |  | No | No | No | No |
| 35 | Dominica | UMIC |  | Yes | No | Yes | No |
| 36 | Dominican Republic | UMIC |  | Yes | Yes | Yes | Yes |
| 37 | Ecuador | UMIC |  | Yes | No | No | No |
| 38 | Egypt, Arab Rep. | LMIC | Strong | Yes | No | Yes | No |
| 39 | El Salvador | LMIC |  | Yes | Yes | Yes | Yes |
| 40 | Eritrea | LIC |  | No | No | No | No |
| 41 | Ethiopia | LIC | Medium | Yes | Yes | Yes | Yes |
| 42 | Fiji | UMIC |  | Yes | Yes | Yes | Yes |
| 43 | Gabon | UMIC |  | No | No | No | No |
| 44 | Gambia, The | LIC |  | Yes | Yes | No | No |
| 45 | Georgia | LMIC |  | Yes | Yes | Yes | Yes |
| 46 | Ghana | LMIC | Weak | Yes | Yes | No | No |
| 47 | Grenada | UMIC |  | Yes | Yes | Yes | Yes |
| 48 | Guatemala | LMIC | Medium | Yes | Yes | Yes | Yes |
| 49 | Guinea | LIC |  | Yes | Yes | Yes | Yes |
| 50 | Guinea-Bissau | LIC |  | Yes | No | No | No |
| 51 | Guyana | LMIC |  | No | No | No | No |
| 52 | Haiti | LIC |  | Yes | Yes | Yes | Yes |
| 53 | Honduras | LMIC |  | Yes | Yes | No | No |
| 54 | Hungary | UMIC |  | No | No | Yes | Yes |
| 55 | India | LMIC | Strong | Yes | Yes | No | No |
| 56 | Indonesia | LMIC | Medium | Yes | Yes | Yes | Yes |
| 57 | Iran, Islamic Rep. | UMIC |  | No | No | No | No |
| 58 | Iraq | UMIC | Weak | Yes | Yes | Yes | Yes |
| 59 | Jamaica | UMIC |  | No | No | No | No |
| 60 | Jordan | UMIC |  | No | No | No | No |
| 61 | Kazakhstan | UMIC |  | Yes | Yes | Yes | Yes |
| 62 | Kenya | LIC | Weak | Yes | Yes | Yes | Yes |
| 63 | Kiribati | LMIC |  | Yes | Yes | Yes | Yes |
| 64 | Korea, Dem. Rep. | LIC |  | No | No | No | No |
| 65 | Kosovo | LMIC |  | No | No | No | No |
| 66 | Kyrgyz Republic | LIC |  | Yes | Yes | Yes | Yes |
| 67 | Lao PDR | LMIC |  | Yes | Yes | No | No |
| 68 | Lebanon | UMIC |  | No | No | No | No |
| 69 | Lesotho | LMIC |  | No | No | No | No |
| 70 | Liberia | LIC |  | Yes | Yes | No | No |
| 71 | Libya | UMIC |  | No | No | No | No |
| 72 | Macedonia, FYR | UMIC |  | No | No | No | No |
| 73 | Madagascar | LIC | Medium | Yes | Yes | Yes | Yes |
| 74 | Malawi | LIC | Strong | Yes | Yes | No | No |
| 75 | Malaysia | UMIC |  | Yes | Yes | Yes | Yes |
| 76 | Maldives | UMIC |  | Yes | Yes | Yes | Yes |
| 77 | Mali | LIC | Weak | Yes | Yes | Yes | Yes |
| 78 | Marshall Islands | UMIC |  | No | No | No | No |
| 79 | Mauritania | LMIC |  | Yes | Yes | Yes | Yes |
| 80 | Mauritius | UMIC |  | Yes | Yes | Yes | Yes |
| 81 | Mexico | UMIC |  | Yes | Yes | Yes | Yes |
| 82 | Micronesia, Fed. Sts. | LMIC |  | No | No | No | No |
| 83 | Moldova | LMIC |  | No | No | No | No |
| 84 | Mongolia | LMIC |  | Yes | Yes | Yes | Yes |
| 85 | Montenegro | UMIC |  | No | No | No | No |
| 86 | Morocco | LMIC |  | Yes | No | Yes | Yes |
| 87 | Mozambique | LIC | Weak | Yes | Yes | No | No |
| 88 | Myanmar | LIC | Medium | Yes | No | No | No |
| 89 | Namibia | UMIC |  | Yes | Yes | Yes | Yes |
| 90 | Nepal | LIC | Medium | Yes | Yes | No | No |
| 91 | Nicaragua | LMIC |  | Yes | Yes | Yes | Yes |
| 92 | Niger | LIC | Medium | No | No | No | No |
| 93 | Nigeria | LMIC | Strong | Yes | Yes | Yes | Yes |
| 94 | Pakistan | LMIC | Weak | Yes | No | No | No |
| 95 | Palau | UMIC |  | No | No | No | No |
| 96 | Panama | UMIC |  | No | No | No | No |
| 97 | Papua New Guinea | LMIC |  | Yes | No | No | No |
| 98 | Paraguay | LMIC |  | No | No | No | No |
| 99 | Peru | UMIC | Strong | Yes | Yes | No | No |
| 100 | Philippines | LMIC | Strong | Yes | Yes | Yes | Yes |
| 101 | Romania | UMIC |  | No | No | Yes | No |
| 102 | Rwanda | LIC |  | Yes | Yes | Yes | Yes |
| 103 | Samoa | LMIC |  | No | No | No | No |
| 104 | Sao Tome and Principe | LMIC |  | No | No | No | No |
| 105 | Senegal | LMIC |  | Yes | Yes | No | No |
| 106 | Serbia | UMIC |  | No | No | No | No |
| 107 | Seychelles | UMIC |  | No | No | Yes | Yes |
| 108 | Sierra Leone | LIC |  | Yes | Yes | Yes | Yes |
| 109 | Solomon Islands | LMIC |  | Yes | Yes | Yes | Yes |
| 110 | Somalia | LIC |  | No | No | No | No |
| 111 | South Africa | UMIC | Medium | Yes | Yes | Yes | Yes |
| 112 | South Sudan | LIC |  | Yes | Yes | No | No |
| 113 | Sri Lanka | LMIC |  | Yes | Yes | Yes | Yes |
| 114 | St. Lucia | UMIC |  | No | No | No | No |
| 115 | St. Vincent and the Grenadines | UMIC |  | No | No | No | No |
| 116 | Sudan | LMIC | Medium | Yes | Yes | Yes | Yes |
| 117 | Suriname | UMIC |  | No | No | Yes | Yes |
| 118 | Swaziland | LMIC |  | Yes | Yes | Yes | Yes |
| 119 | Syrian Arab Republic | LMIC |  | Yes | No | No | No |
| 120 | Tajikistan | LIC |  | Yes | Yes | No | No |
| 121 | Tanzania | LIC | Weak | Yes | Yes | No | No |
| 122 | Thailand | UMIC |  | Yes | Yes | Yes | Yes |
| 123 | Timor-Leste | LMIC |  | Yes | Yes | No | No |
| 124 | Togo | LIC |  | Yes | Yes | Yes | Yes |
| 125 | Tonga | UMIC |  | Yes | No | No | No |
| 126 | Tunisia | UMIC |  | Yes | Yes | Yes | No |
| 127 | Turkey | UMIC | Medium | No | No | Yes | Yes |
| 128 | Turkmenistan | UMIC |  | No | No | No | No |
| 129 | Tuvalu | UMIC |  | No | No | Yes | Yes |
| 130 | Uganda | LIC | Weak | Yes | Yes | No | No |
| 131 | Ukraine | LMIC |  | No | No | Yes | Yes |
| 132 | Uzbekistan | LMIC |  | No | No | Yes | Yes |
| 133 | Vanuatu | LMIC |  | No | No | No | No |
| 134 | Venezuela, RB | UMIC |  | No | No | No | No |
| 135 | Vietnam | LMIC | Strong | Yes | Yes | Yes | Yes |
| 136 | West Bank and Gaza | LMIC |  | Yes | Yes | Yes | Yes |
| 137 | Yemen, Rep. | LMIC | Weak | Yes | Yes | No | No |
| 138 | Zambia | LMIC | Medium | Yes | Yes | Yes | Yes |
| 139 | Zimbabwe | LIC |  | Yes | Yes | Yes | Yes |
