# Supplementary material for: Strong nutrition governance is a key to addressing nutrition transition in low and middle-income countries: review of countries’ nutrition policies
Source: Nutr J. 2014 Jun 27;13:65. doi: 10.1186/1475-2891-13-65 (PMC4110371; doi:10.1186/1475-2891-13-65)
Supplement: Additional file 2: Table S2 — Data on country, regional distribution, policy, nutrition status. [file 1475-2891-13-65-S2.doc]

| **Additional file 2: Data on country, regional distribution, policy, nutrition status** | | | | | | | | | | | | | |
| --- | --- | --- | --- | --- | --- | --- | --- | --- | --- | --- | --- | --- | --- |
| **No** | **Country name** | **Income level** | **Region** | **Policy-Undernutrition** | **Policy-Overweight** | **Latest Stunting** | **Year Range** | **Latest Wasting** | **Year Range** | **Latest Underweight** | **Year Range** | **Latest Overweight** | **Year Range** |
| 1 | Afghanistan | Low | South Asia | YES | NO | 59.3 | 1997-2004 | 8.6 | 1997-2004 | 32.9 | 1997-2004 | 4.6 | 1997-2004 |
| 2 | Albania | UM | Europe and Central Asia | NO | NO | 23.1 | 2000-2009 | 9.4 | 2000-2009 | 6.3 | 2000-2009 | 23.4 | 2000-2009 |
| 3 | Algeria | UM | Middle East and North Africa | YES | NO | 15.9 | 2000-2005 | 4.0 | 2000-2005 | 3.7 | 2000-2005 | 12.9 | 2000-2005 |
| 4 | American Samoa | UM | East Asia Pacific | NO | NO |  |  |  |  |  |  |  |  |
| 5 | Angola | UM | Sub Saharan Africa | NO | NO | 29.2 | 1996-2007 | 8.2 | 1996-2007 | 15.6 | 1996-2007 |  | 1996-2007 |
| 6 | Argentina | UM | Latin America and Caribbean | YES | NO | 8.2 | 1994-2005 | 1.2 | 1994-2005 | 2.3 | 1994-2005 | 9.9 | 1994-2005 |
| 7 | Armenia | LM | Latin America and Caribbean | YES | NO | 20.8 | 2000-2010 | 4.2 | 2000-2010 | 5.3 | 2000-2010 | 16.8 | 2000-2010 |
| 8 | Azerbaijan | UM | Europe and Central Asia | YES | NO | 26.8 | 2000-2006 | 6.8 | 2000-2006 | 8.4 | 2000-2006 | 13.9 | 2000-2006 |
| 9 | Bangladesh | Low | South Asia | YES | YES | 41.4 | 2006-2011 | 15.7 | 2006-2011 | 36.8 | 2006-2011 | 1.9 | 2006-2011 |
| 10 | Belarus | UM | Europe and Central Asia | NO | NO | 4.5 | 2005 | 2.2 | 2005 | 1.3 | 2005 | 9.7 | 2005 |
| 11 | Belize | UM | Latin America and Caribbean | YES | NO | 19.3 | 2006-2011 | 3.3 | 2006-2011 | 6.2 | 2006-2011 | 7.9 | 2006-2011 |
| 12 | Benin | Low | Sub Saharan Africa | YES | NO | 44.7 | 1996-2006 | 8.4 | 1996-2006 | 20.2 | 1996-2006 | 11.4 | 1996-2006 |
| 13 | Bhutan | LM | South Asia | YES | NO | 33.6 | 1999-2010 | 5.9 | 1999-2010 | 12.8 | 1999-2010 | 7.6 | 1999-2010 |
| 14 | Bolivia | LM | Latin America and Caribbean | YES | NO | 27.2 | 1998-2008 | 1.4 | 1998-2008 | 4.5 | 1998-2008 | 8.7 | 1998-2008 |
| 15 | Bosnia Herzegovina | UM | Europe and Central Asia | YES | NO | 8.9 | 2000-2012 | 2.3 | 2000-2012 | 1.5 | 2000-2012 | 17.4 | 2000-2012 |
| 16 | Botswana | UM | Sub Saharan Africa | YES | NO | 31.4 | 1996-2008 | 7.2 | 1996-2008 | 11.2 | 1996-2008 | 11.2 | 1996-2008 |
| 17 | Brazil | UM | Latin America and Caribbean | YES | YES | 7.1 | 1996-2007 | 1.6 | 1996-2007 | 2.2 | 1996-2007 | 7.3 | 1996-2007 |
| 18 | Bulgaria | UM | Europe and Central Asia | YES | YES | 8.4 | 2004 | 3.2 | 2004 | 1.6 | 2004 | 13.6 | 2004 |
| 19 | Burkina Faso | Low | Sub Saharan Africa | YES | YES | 35.1 | 2006-2010 | 15.4 | 2006-2010 | 26.2 | 2006-2010 | 2.8 | 2006-2010 |
| 20 | Burundi | Low | Sub Saharan Africa | YES | YES | 57.5 | 2000-2011 | 6.1 | 2000-2011 | 29.1 | 2000-2011 | 2.9 | 2000-2011 |
| 21 | Cambodia | Low | East Asia Pacific | YES | YES | 40.9 | 2005-2011 | 10.8 | 2005-2011 | 29 | 2005-2011 | 1.9 | 2005-2011 |
| 22 | Cameroon | LM | Sub Saharan Africa | YES | NO | 32.6 | 2004-2011 | 5.8 | 2004-2011 | 15.1 | 2004-2011 | 6.5 | 2004-2011 |
| 23 | Cape Verde | LM | Sub Saharan Africa | YES | NO | 21.4 | 1983-1994 | 6.9 | 1983-1994 | 11.8 | 1983-1994 |  | 1983-1994 |
| 24 | Central African Republic | Low | Sub Saharan Africa | YES | NO | 45.1 | 1995-2006 | 12.2 | 1995-2006 | 28 | 1995-2006 | 8.5 | 1995-2006 |
| 25 | Chad | Low | Sub Saharan Africa | YES | NO | 44.8 | 1996-2004 | 16.1 | 1996-2004 | 33.9 | 1996-2004 | 4.4 | 1996-2004 |
| 26 | China | UM | East Asia Pacific | YES | YES | 9.4 | 2008-2010 | 2.3 | 2008-2010 | 3.4 | 2008-2010 | 6.6 | 2008-2010 |
| 27 | Colombia | UM | Latin America and Caribbean | YES | YES | 12.7 | 2000-2010 | 0.9 | 2000-2010 | 3.4 | 2000-2010 | 4.8 | 2000-2010 |
| 28 | Comoros | Low | Sub Saharan Africa | NO | NO | 46.9 | 1991-2000 | 13.3 | 1991-2000 | 25 | 1991-2000 | 21.5 | 1991-2000 |
| 29 | Congo, Dem. Rep. | Low | Sub Saharan Africa | YES | YES | 43.5 | 2001-2010 | 8.5 | 2001-2010 | 24.2 | 2001-2010 | 4.9 | 2001-2010 |
| 30 | Congo, Rep. | LM | Sub Saharan Africa | YES | NO | 31.2 | 1987-2005 | 8 | 1987-2005 | 11.8 | 1987-2005 | 8.5 | 1987-2005 |
| 31 | Costa Rica | UM | Latin America and Caribbean | YES | YES | 5.6 | 1996-2009 | 1 | 1996-2009 | 1.1 | 1996-2009 | 8.1 | 1996-2009 |
| 32 | Cote d'Ivoire | LM | Sub Saharan Africa | YES | YES | 39 | 1997-2008 | 14 | 1997-2008 | 29.4 | 1997-2008 | 4.9 | 1997-2008 |
| 33 | Cuba | UM | Latin America and Caribbean | YES | NO | 7 | 2000 | 2.4 | 2000 | 3.4 | 2000 |  | 2000 |
| 34 | Djibouti | LM | Middle East and North Africa | NO | NO | 33.5 | 2002-2012 | 21.5 | 2002-2012 | 29.8 | 2002-2012 | 8.1 | 2002-2012 |
| 35 | Dominica | UM | Latin America and Caribbean | YES | YES |  |  |  |  |  |  |  |  |
| 36 | Dominican Republic | UM | Latin America and Caribbean | YES | YES | 10.1 | 2002-2007 | 2.3 | 2002-2007 | 3.4 | 2002-2007 | 8.3 | 2002-2007 |
| 37 | Ecuador | UM | Latin America and Caribbean | YES | NO | 29 | 1986-2004 | 2.3 | 1986-2004 | 6.2 | 1986-2004 | 5.1 | 1986-2004 |
| 38 | Egypt, Arab Rep. | LM | Middle East and North Africa | YES | YES | 30.7 | 2003-2008 | 7.9 | 2003-2008 | 6.8 | 2003-2008 | 20.5 | 2003-2008 |
| 39 | El Salvador | LM | Latin America and Caribbean | YES | YES | 20.6 | 1998-2008 | 1.6 | 1998-2008 | 6.6 | 1998-2008 | 5.7 | 1998-2008 |
| 40 | Eritrea | Low | Sub Saharan Africa | NO | NO | 43.7 | 1993-2002 | 14.9 | 1993-2002 | 34.5 | 1993-2002 | 1.6 | 1993-2002 |
| 41 | Ethiopia | Low | Sub Saharan Africa | YES | YES | 44.2 | 2000-2011 | 10.1 | 2000-2011 | 29.2 | 2000-2011 | 1.8 | 2000-2011 |
| 42 | Fiji | UM | East Asia Pacific | YES | YES | 7.5 | 1993-2004 | 6.3 | 1993-2004 | 5.3 | 1993-2004 | 5.1 | 1993-2004 |
| 43 | Gabon | UM | Sub Saharan Africa | NO | NO | 17.5 | 2000-2012 | 3.4 | 2000-2012 | 6.5 | 2000-2012 | 7.7 | 2000-2012 |
| 44 | Gambia, The | Low | Sub Saharan Africa | YES | NO | 27.6 | 1996-2006 | 7.4 | 1996-2006 | 15.8 | 1996-2006 | 2.7 | 1996-2006 |
| 45 | Georgia | LM | Europe and Central Asia | YES | YES | 11.3 | 1999-2009 | 1.6 | 1999-2009 | 1.1 | 1999-2009 | 19.9 | 1999-2009 |
| 46 | Ghana | LM | Sub Saharan Africa | YES | NO | 28.6 | 2003-2008 | 8.7 | 2003-2008 | 14.3 | 2003-2008 | 5.8 | 2003-2008 |
| 47 | Grenada | UM | Latin America and Caribbean | YES | YES |  |  |  |  |  |  |  |  |
| 48 | Guatemala | LM | Latin America and Caribbean | YES | YES | 48 | 2000-2009 | 1.1 | 2000-2009 | 13 | 2000-2009 | 4.9 | 2000-2009 |
| 49 | Guinea | Low | Sub Saharan Africa | YES | YES | 35.8 | 2005-2012 | 5.6 | 2005-2012 | 16.3 | 2005-2012 | 3.1 | 2005-2012 |
| 50 | Guinea-Bissau | Low | Sub Saharan Africa | YES | NO | 27.7 | 2000-2008 | 4.8 | 2000-2008 | 16.6 | 2000-2008 | 2.2 | 2000-2008 |
| 51 | Guyana | LM | Latin America and Caribbean | NO | NO | 19.5 | 2000-2009 | 5.3 | 2000-2009 | 11.1 | 2000-2009 | 6.7 | 2000-2009 |
| 52 | Haiti | Low | Latin America and Caribbean | YES | YES | 29.7 | 1994-2006 | 10.3 | 1994-2006 | 18.9 | 1994-2006 | 3.9 | 1994-2006 |
| 53 | Honduras | LM | Latin America and Caribbean | YES | NO | 29.9 | 1996-2006 | 1.4 | 1996-2006 | 8.6 | 1996-2006 | 5.8 | 1996-2006 |
| 54 | Hungary | UM | Europe | NO | YES | 3.3 | 1980-1988 | 4.2 | 1980-1988 | 2.3 | 1980-1988 | 3 | 1980-1988 |
| 55 | India | LM | South Asia | YES | NO | 47.9 | 1996-2006 | 20 | 1996-2006 | 43.5 | 1996-2006 | 1.9 | 1996-2006 |
| 56 | Indonesia | LM | East Asia Pacific | YES | YES | 39.2 | 2007-2010 | 12.3 | 2007-2010 | 18.6 | 2007-2010 | 12.3 | 2007-2010 |
| 57 | Iran, Islamic Rep. | UM | Middle East and North Africa | NO | NO | 7.1 | 1995-2004 | 4.8 | 1995-2004 | 4.6 | 1995-2004 |  | 1995-2004 |
| 58 | Iraq | UM | Middle East and North Africa | YES | YES | 27.5 | 2003-2006 | 5.8 | 2003-2006 | 7.1 | 2003-2006 | 15 | 2003-2006 |
| 59 | Jamaica | UM | Latin America and Caribbean | NO | NO | 4.8 | 2007-2010 | 3.1 | 2007-2010 | 3.2 | 2007-2010 | 4 | 2007-2010 |
| 60 | Jordan | UM | Middle East and North Africa | NO | NO | 8.3 | 1997-2009 | 1.6 | 1997-2009 | 1.9 | 1997-2009 | 6.6 | 1997-2009 |
| 61 | Kazakhstan | UM | Europe and Central Asia | YES | YES | 13.1 | 1999-2011 | 4.1 | 1999-2011 | 3.7 | 1999-2011 | 13.3 | 1999-2011 |
| 62 | Kenya | Low | Sub Saharan Africa | YES | YES | 35.2 | 2003-2009 | 7 | 2003-2009 | 16.4 | 2003-2009 | 5 | 2003-2009 |
| 63 | Kiribati | LM | East Asia Pacific | YES | YES | 34.4 | 1985 | 12.6 | 1985 | 11.3 | 1985 | 15.9 | 1985 |
| 64 | Korea, Dem. Rep. | Low | East Asia Pacific | NO | NO | 32.4 | 2002-2009 | 5.2 | 2002-2009 | 18.8 | 2002-2009 | 0 | 2002-2009 |
| 65 | Kosovo | LM | Europe and Central Asia | NO | NO |  |  |  |  |  |  |  |  |
| 66 | Kyrgyz Republic | Low | Europe and Central Asia | YES | YES | 18.1 | 1997-2006 | 3.4 | 1997-2006 | 2.7 | 1997-2006 | 8.7 | 1997-2006 |
| 67 | Lao PDR | LM | East Asia Pacific | YES | NO | 47.6 | 1994-2006 | 7.3 | 1994-2006 | 31.6 | 1994-2006 | 1.3 | 1994-2006 |
| 68 | Lebanon | UM | Middle East and North Africa | NO | NO | 16.5 | 1997-2004 | 6.6 | 1997-2004 | 4.2 | 1997-2004 | 6.2 | 1997-2004 |
| 69 | Lesotho | LM | Sub Saharan Africa | NO | NO | 39 | 2000-2010 | 3.9 | 2000-2010 | 13.5 | 2000-2010 | 13.5 | 2000-2010 |
| 70 | Liberia | Low | Sub Saharan Africa | YES | NO | 39.4 | 1976-2007 | 7.8 | 1976-2007 | 20.4 | 1976-2007 | 4.2 | 1976-2007 |
| 71 | Libya | UM | Middle East and North Africa | NO | NO | 21 | 1995-2007 | 6.5 | 1995-2007 | 5.6 | 1995-2007 | 22.4 | 1995-2007 |
| 72 | Macedonia, FYR | UM | Europe and Central Asia | NO | NO | 11.5 | 1999-2005 | 3.4 | 1999-2005 | 1.8 | 1999-2005 | 16.2 | 1999-2005 |
| 73 | Madagascar | Low | Sub Saharan Africa | YES | YES | 49.2 | 1997-2009 | 15.2 | 1997-2004 | 36.8 | 1997-2004 | 6.2 | 1997-2004 |
| 74 | Malawi | Low | Sub Saharan Africa | YES | NO | 47.8 | 2006-2010 | 4.1 | 2006-2010 | 13.8 | 2006-2010 | 9.2 | 2006-2010 |
| 75 | Malaysia | UM | East Asia Pacific | YES | YES | 17.2 | 1999-2006 | 15.3 | 1999 | 12.9 | 1995-2006 | 5.5 | 1999 |
| 76 | Maldives | UM | South Asia | YES | YES | 20.3 | 1997-2009 | 10.2 | 1997-2009 | 17.8 | 1997-2009 | 6.5 | 1997-2009 |
| 77 | Mali | Low | Sub Saharan Africa | YES | YES | 38.5 | 1996-2006 | 15.3 | 1996-2006 | 27.9 | 1996-2006 | 4.7 | 1996-2006 |
| 78 | Marshall Islands | UM | East Asia Pacific | NO | NO |  |  |  |  |  |  |  |  |
| 79 | Mauritania | LM | Sub Saharan Africa | YES | YES | 22 | 2007-2012 | 11.6 | 2007-2012 | 19.5 | 2007-2012 | 1.2 | 2007-2012 |
| 80 | Mauritius | UM | Sub Saharan Africa | YES | YES | 13.6 | 1985-1995 | 15.7 | 1985-1995 | 13 | 1985-1995 | 6.5 | 1985-1995 |
| 81 | Mexico | UM | Latin America and Caribbean | YES | YES | 13.6 | 1998-2012 | 1.6 | 1998-2012 | 2.8 | 1998-2012 | 9 | 1998-2012 |
| 82 | Micronesia, Fed. Sts. | LM | East Asia Pacific | NO | NO |  |  |  |  |  |  |  |  |
| 83 | Moldova | LM | Europe and Central Asia | NO | NO |  |  |  |  |  |  |  |  |
| 84 | Mongolia | LM | East Asia Pacific | YES | YES | 27.5 | 2000-2005 | 2.7 | 2000-2005 | 5.3 | 2000-2005 | 14.2 | 2000-2005 |
| 85 | Montenegro | UM | Europe and Central Asia | NO | NO | 7.9 | 2005-2006 | 4.2 | 2005-2006 | 2.2 | 2005-2006 | 15.6 | 2005-2006 |
| 86 | Morocco | LM | Middle East and North Africa | YES | YES | 14.9 | 1996-2011 | 2.3 | 1996-2011 | 3.1 | 1996-2011 | 10.7 | 1996-2011 |
| 87 | Mozambique | Low | Sub Saharan Africa | YES | NO | 43.1 | 2003-2011 | 6.1 | 2003-2011 | 15.6 | 2003-2011 | 7.9 | 2003-2011 |
| 88 | Myanmar | Low | East Asia Pacific | YES | NO | 35.1 | 2000-2010 | 7.9 | 2000-2010 | 22.6 | 2000-2010 | 2.6 | 2000-2010 |
| 89 | Namibia | UM | Sub Saharan Africa | YES | YES | 29.6 | 1992-2007 | 7.5 | 1992-2007 | 17.5 | 1992-2007 | 4.6 | 1992-2007 |
| 90 | Nepal | Low | South Asia | YES | NO | 40.5 | 2001-2011 | 11.2 | 2001-2011 | 29.1 | 2001-2011 | 1.5 | 2001-2011 |
| 91 | Nicaragua | LM | Latin America and Caribbean | YES | YES | 23 | 2001-2007 | 1.5 | 2001-2007 | 0 | 2001-2007 | 6.3 | 2001-2007 |
| 92 | Niger | Low | Sub Saharan Africa | NO | NO | 54.8 | 1998-2002 | 12.4 | 1998-2002 | 39.9 | 1998-2002 | 3.5 | 1998-2002 |
| 93 | Nigeria | LM | Sub Saharan Africa | YES | YES | 36 | 2007-2011 | 10.2 | 2007-2011 | 24.4 | 2007-2011 | 3 | 2007-2011 |
| 94 | Pakistan | LM | South Asia | YES | NO | 43 | 2001-2011 | 14.8 | 2001-2011 | 30.9 | 2001-2011 | 6.4 | 2001-2011 |
| 95 | Palau | UM | East Asia Pacific | NO | NO |  |  |  |  |  |  |  |  |
| 96 | Panama | UM | Latin America and Caribbean | NO | NO | 19.1 | 1997-2008 | 1.2 | 1997-2008 | 3.9 | 1997-2008 | 6.2 | 1997 |
| 97 | Papua New Guinea | LM | East Asia Pacific | YES | NO | 43.9 | 1983-2005 | 4.4 | 1983-2005 | 18.1 | 1983-2005 | 3.4 | 1983-2005 |
| 98 | Paraguay | LM | Latin America and Caribbean | NO | NO | 17.5 | 1990-2005 | 1.1 | 1990-2005 | 3.4 | 1990-2005 | 7.9 | 1990-2005 |
| 99 | Peru | UM | Latin America and Caribbean | YES | NO | 28.3 | 2000-2008 | 0.8 | 2000-2008 | 4.5 | 2000-2008 | 9.8 | 2000-2008 |
| 100 | Philippines | LM | East Asia Pacific | YES | YES | 33.6 | 2003-2011 | 7.3 | 2003-2011 | 20.2 | 2003-2011 | 4.3 | 2003-2011 |
| 101 | Romania | UM | Europe and Central Asia | NO | YES | 12.8 | 2000-2002 | 3.5 | 2000-2002 | 3.5 | 2000-2002 | 8.3 | 2000-2002 |
| 102 | Rwanda | Low | Sub Saharan Africa | YES | YES | 44.3 | 2000-2011 | 3 | 2000-2011 | 11.7 | 2000-2011 | 7.1 | 2000-2011 |
| 103 | Samoa | LM | East Asia Pacific | NO | NO | 6.4 | 2009 | 1.3 | 2009 | 1.7 | 2009 | 6.2 | 2009 |
| 104 | Sao Tome and Principe | LM | Sub Saharan Africa | NO | NO | 31.6 | 2000-2009 | 11.2 | 2000-2009 | 14.4 | 2000-2009 | 11.6 | 2000-2009 |
| 105 | Senegal | LM | Sub Saharan Africa | YES | NO | 15.5 | 2005-2012 | 8.7 | 2005-2012 | 14.4 | 2005-2012 | 0.7 | 2005-2012 |
| 106 | Serbia | UM | Europe and Central Asia | NO | NO | 6.6 | 2006-2010 | 3.5 | 2006-2010 | 1.6 | 2006-2010 | 15.6 | 2006-2010 |
| 107 | Seychelles | UM | Sub Saharan Africa | NO | YES | 7.7 | 1988 | 2.7 | 1988 | 5 | 1988 | 5.8 | 1988 |
| 108 | Sierra Leone | Low | Sub Saharan Africa | YES | YES | 32.6 | 2008-2010 | 7.6 | 2008-2010 | 21.1 | 2008-2010 | 1.4 | 2008-2010 |
| 109 | Solomon Islands | LM | East Asia Pacific | YES | YES | 32.8 | 1970-2007 | 4.3 | 1970-2007 | 11.5 | 1970-2007 | 2.5 | 1970-2007 |
| 110 | Somalia | Low | Sub Saharan Africa | NO | NO | 42.1 | 2000-2006 | 13.2 | 2000-2006 | 32.8 | 2000-2006 | 4.7 | 2000-2006 |
| 111 | South Africa | UM | Sub Saharan Africa | YES | YES | 23.9 | 1999-2008 | 4.7 | 1999-2008 | 8.7 | 1999-2008 | 19.2 | 1999- |
| 112 | South Sudan | Low | Sub Saharan Africa | YES | NO | 36.2 | 2006 | 26.6 | 2006 | 32.5 | 2006 | 10.9 | 2006 |
| 113 | Sri Lanka | LM | South Asia | YES | YES | 19.2 | 2000-2009 | 11.8 | 2000-2009 | 21.6 | 2000-2009 | 0.8 | 2000-2009 |
| 114 | St. Lucia | UM | Latin America and Caribbean | NO | NO |  |  |  |  |  |  |  |  |
| 115 | St. Vincent and the Grenadines | UM | Latin America and Caribbean | NO | NO |  |  |  |  |  |  |  |  |
| 116 | Sudan | LM | Sub Saharan Africa | YES | YES | 38.3 | 2006 | 14.5 | 2006 | 27 | 2006 | 4.2 | 2006 |
| 117 | Suriname | UM | Latin America and Caribbean | NO | YES | 8.8 | 2000-2010 | 5 | 2000-2010 | 5.8 | 2000-2010 | 4 | 2000-2010 |
| 118 | Swaziland | LM | Sub Saharan Africa | YES | YES | 31 | 2007-2010 | 0.8 | 2007-2010 | 5.8 | 2007-2010 | 10.7 | 2007-2010 |
| 119 | Syrian Arab Republic | LM | Middle East and North Africa | YES | NO | 27.5 | 2001-2009 | 11.5 | 2001-2009 | 10.1 | 2001-2009 | 17.9 | 2001-2009 |
| 120 | Tajikistan | Low | Europe and Central Asia | YES | NO | 39.2 | 2003-2007 | 6.7 | 2003-2007 | 15 | 2003-2007 |  | 2003-2007 |
| 121 | Tanzania | Low | Sub Saharan Africa | YES | NO | 42.5 | 1999-2010 | 4.9 | 1999-2010 | 16.2 | 1999-2010 | 5.5 | 1999-2010 |
| 122 | Thailand | UM | East Asia Pacific | YES | YES | 15.7 | 1993-2006 | 4.7 | 1993-2006 | 7 | 1993-2006 | 8 | 1993-2006 |
| 123 | Timor-Leste | LM | East Asia Pacific | YES | NO | 57.7 | 2003-2010 | 18.9 | 2003-2010 | 45.3 | 2003-2010 | 5.8 | 2003-2010 |
| 124 | Togo | Low | Sub Saharan Africa | YES | YES | 29.8 | 2006-2010 | 4.8 | 2006-2010 | 16.5 | 2006-2010 | 1.6 | 2006-2010 |
| 125 | Tonga | UM | East Asia Pacific | YES | NO | 2.2 | 1986- | 1.3 | 1986- |  |  |  |  |
| 126 | Tunisia | UM | Middle East and North Africa | YES | YES | 9 | 1997-2006 | 3.4 | 1997-2006 | 3.3 | 1997-2006 | 8.8 | 1997-2006 |
| 127 | Turkey | UM | Europe and Central Asia | NO | YES | 15.6 | 1998-2004 | 1.1 | 1998-2004 | 3.5 | 1998-2004 | 9.1 | 1998-2004 |
| 128 | Turkmenistan | UM | Europe and Central Asia | NO | NO | 28.1 | 2000 | 7.1 | 2000 | 10.5 | 2000 |  |  |
| 129 | Tuvalu | UM | East Asia Pacific | NO | YES | 10 | 2007 | 3.3 | 2007 | 1.6 | 2007 | 6.3 | 2007 |
| 130 | Uganda | Low | Sub Saharan Africa | YES | NO | 33.7 | 2001-2011 | 4.8 | 2001-2011 | 14.1 | 2001-2011 | 3.8 | 2001-2011 |
| 131 | Ukraine | LM | Europe and Central Asia | NO | YES | 4.3 | 2000-2002 | 0.3 | 2000-2002 | 0.9 | 2000-2002 | 26.5 | 2000-2002 |
| 132 | Uzbekistan | LM | Europe and Central Asia | NO | YES | 19.6 | 1996-2006 | 4.5 | 1996-2006 | 4.4 | 1996-2006 | 12.8 | 1996-2006 |
| 133 | Vanuatu | LM | East Asia Pacific | NO | NO | 25.9 | 1983-2007 | 5.9 | 1983-2007 | 11.7 | 1983-2007 | 4.7 | 1983-2007 |
| 134 | Venezuela, RB | UM | Latin America and Caribbean | NO | NO | 13.4 | 2007-2009 | 4.1 | 2007-2009 | 2.9 | 2007-2009 | 6.4 | 2007-2009 |
| 135 | Vietnam | LM | East Asia Pacific | YES | YES | 23.3 | 2007-2011 | 4.4 | 2007-2011 | 12 | 2007-2011 | 4.6 | 2007-2011 |
| 136 | West Bank and Gaza | LM | Middle East and North Africa | YES | YES |  |  |  |  |  |  |  |  |
| 137 | Yemen, Rep. | LM | Middle East and North Africa | YES | NO | 51.7 | 1996-2003 | 15.2 | 1996-2003 | 43.1 | 1996-2003 | 5 | 1996-2003 |
| 138 | Zambia | LM | Sub Saharan Africa | YES | YES | 45.8 | 1999-2007 | 5.6 | 1999-2007 | 14.9 | 1999-2007 | 8.4 | 1999-2007 |
| 139 | Zimbabwe | Low | Sub Saharan Africa | YES | YES | 32.3 | 1999-2011 | 3.1 | 1999-2011 | 10.1 | 1999-2011 | 5.8 | 1999-2011 |
